# Continuous Electrical Currents in the Treatment of the Suspension of Vital Actions Caused by Chloroform

**Published:** 1869-01

**Authors:** 


CONTINUOUS ELECTRICAL CURRENTS IN THE TREATMENT OF THE SUSPENSION OF VITAL ACTIONS CAUSED BY CHLOROFORM. —MM. Onimus and Legros, after examining the effects of constant electrical currents on the heart and its nerves, were led to believe that such currents might prove efficient in stimulating the heart's action after its paralysis by chloroform inhalation. They have, accordingly, carefully investigated the subject (*Comtes Rendus*, Mars 9, 1867). They assert that in chloroform syncope there is more or less paralysis of the muscular fibres of the heart. The means hitherto recommended to treat this condition, such as artificial respiration, flagellation, and aspersion with cold water, are insufficient, as they do not directly influence the muscular action of the heart. Interrupted currents of electricity should not be used, as they diminish and even stop the respiratory and cardiac movements. The value of continuous electric currents was tested by experiments on dogs, rabbits, rats and frogs, in the following manner: A rat was placed under a glass cover along with a sponge saturated with chloroform. Its respirations gradually became jerking, and in one minute they had nearly ceased, while the animal was now completely anæsthetized. It was left for thirty seconds longer under the glass cover, and after being withdrawn, it was left untouched for another thirty seconds. No cardiac action was now perceptible. A continuous electric current was then passed from the rectum to the mouth; nothing was observed for several seconds, when the hearts beat reappeared, and then imperfect respiratory movements occurred, which, by the by, became quite normal. The electrolization was now stopped and the animal gradually recovered. Even when left for two minutes in a state of apparent death, the application of a continuous current resuscitated the animal. If an interrupted current were employed in place of a continuous one, death always occurred; but if the former had been employed for only a short time, life could still be restored by the use of a continuous current. The experiments on frogs were of great interest, as the various stages of the effects could be distinctly recognized, especially if the heart were previously exposed. As the exhibition of the anæsthetic was continued the beats diminished in force and number and then ceased; if a continuous current was now used, the beats recommenced. The frog was left to itself for twenty-four hours after complete chloroform anæsthesia; the heart was then quite immobile, and although a continuous electric current could not cause any contractions of the voluntary muscles, it caused a renewal of the heart's action.—*Journal of Anatomy and Physiology.*

---

ODONTOMES.—M. Broca, the distinguished surgeon and physiologist, has just elucidated the pathology of the follicles of the teeth, the normal evolution of which had already been described in works on histology. M. Broca does not think that the deviations from this normal evolution give rise to peculiar products, but only to tumors made up of the general hypertrophy of the dental substance These tumors, to which the author gives the name of "odontomes," present two forms: some always remain in the state of more or less soft tumors; whilst others, either wholly or in part, assume the hardness of teeth, producing shapeless, irregular dental masses, sometimes growing to a very large size. In fact, any tumor formed from one or more of the substances entering into the formation of a tooth is due to the dentification of a soft tumor of the same form and volume which originally contained hypertrophied odontogenic tissues. This hypertrophied tumor stands in the same connection, with regard to the dentified tumer, as the normal dental bulb does to the healthy tooth.—*Lancet.*

